# No add‐on effect of tDCS on fatigue and depression in chronic stroke patients: A randomized sham‐controlled trial combining tDCS with computerized cognitive training

**DOI:** 10.1002/brb3.2643

**Published:** 2022-06-06

**Authors:** Kristine M. Ulrichsen, Knut K. Kolskår, Geneviève Richard, Mads Lund Pedersen, Dag Alnæs, Erlend S. Dørum, Anne‐Marthe Sanders, Sveinung Tornås, Luigi A. Maglanoc, Andreas Engvig, Hege Ihle‐Hansen, Jan E. Nordvik, Lars T. Westlye

**Affiliations:** ^1^ NORMENT, Division of Mental Health and Addiction, Oslo University Hospital & Institute of Clinical Medicine University of Oslo Oslo Norway; ^2^ Department of Psychology University of Oslo Oslo Norway; ^3^ Sunnaas Rehabilitation Hospital HT Nesodden Norway; ^4^ Bjørknes College Oslo Norway; ^5^ University Center for Information Technology University of Oslo Oslo Norway; ^6^ Department of Medicine Diakonhjemmet Hospital Oslo Norway; ^7^ Department of Nephrology Oslo University Hospital Oslo Norway; ^8^ Department of Neurology Oslo University Hospital Oslo Norway; ^9^ CatoSenteret Rehabilitation Center Son Norway; ^10^ KG Jebsen Centre for Neurodevelopmental Disorders University of Oslo Norway

**Keywords:** brain stimulation, chronic stroke, poststroke fatigue, rehabilitation, tDCS

## Abstract

**Background:**

Fatigue and emotional distress rank high among self‐reported unmet needs in life after stroke. Transcranial direct current stimulation (tDCS) may have the potential to alleviate these symptoms for some patients, but the acceptability and effects for chronic stroke survivors need to be explored in randomized controlled trials.

**Methods:**

Using a randomized sham‐controlled parallel design, we evaluated whether six sessions of 1 mA tDCS (anodal over F3, cathodal over O2) combined with computerized cognitive training reduced self‐reported symptoms of fatigue and depression. Among the 74 chronic stroke patients enrolled at baseline, 54 patients completed the intervention. Measures of fatigue and depression were collected at five time points spanning a 2 months period.

**Results:**

While symptoms of fatigue and depression were reduced during the course of the intervention, Bayesian analyses provided evidence for no added beneficial effect of tDCS. Less severe baseline symptoms were associated with higher performance improvement in select cognitive tasks, and study withdrawal was higher in patients with more fatigue and younger age. Time‐resolved symptom analyses by a network approach suggested higher centrality of fatigue items (except item 1 and 2) than depression items.

**Conclusion:**

The results reveal no add‐on effect of tDCS on fatigue or depression but support the notion of fatigue as a relevant clinical symptom with possible implications for treatment adherence and response.

## Graphical abstract

Using a randomized sham‐controlled parallel design, we evaluated whether six sessions of tDCS combined with computerized cognitive training reduced self‐reported symptoms of fatigue and depression in chronic stroke patients. Measures of fatigue and depression were collected at five timepoints spanning two months. While symptoms of fatigue and depression were reduced during the course of the intervention, results revealed no add‐on effect of tDCS.

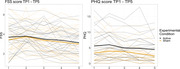

## 1 INTRODUCTION

Although recent years have offered considerable improvements in acute stroke care and survival (Walsh et al., 2015), many stroke survivors experience persistent sequelae (Hankey et al., 2002). Described as a “sense of exhaustion, lack of perceived energy or tiredness, distinct from sadness or weakness” (Leegaard, 1983), poststroke fatigue (PSF) is among the most frequently reported (Walsh et al., 2015) and least understood long‐term consequences of stroke (De Doncker et al., 2018). Fatigue has been shown to increase with time since stroke (Cumming et al., 2018), and a survey of stroke survivors up to 5 years post stroke identified fatigue, emotional problems, and cognitive impairments as the most burdening symptoms (Walsh et al., 2015), indicating a need to target these symptoms in the chronic stroke population.

Generally considered to be a multifactorial condition, PSF is assumed to result from complex interactions between biological, psychological, cognitive, social, and behavioral factors (Aarnes et al., 2020; Chen et al., 2015; Choi‐Kwon & Kim, 2011; Ponchel et al., 2015; Wu et al., 2015). The pathogenesis of PSF has not been established (Nguyen et al., 2019), but it is conceivable that individual factors contribute differently during the various stages of recovery. While early fatigue may be associated with biological factors such as stroke severity (Chen & Marsh, 2018), lesion characteristics (Tang et al., 2014; Tang et al., 2010), and inflammation (Becker, 2016), fatigue in the chronic phase may be more attributable to behavioral and psychological factors (Wu, Mead, et al., 2015). Cognitive impairments (Passier et al., 2011), including attentional deficits (Radman et al., 2012) and reduced processing speed and impaired working memory (Pihlaja et al., 2014), have been reported among PSF patients up to 10 years after stroke (Maaijwee et al., 2015).

The clinical overlap between PSF and poststroke depression (PSD) is substantial (Cumming et al., 2018)—fatigue is both a symptom and a predictor of depression (Douven et al., 2017; van de Port et al., 2007), and depressive symptoms in the acute or subacute phase have been associated with increased risk of PSF at 1–1.5 years post stroke (Passier et al., 2011; Snaphaan et al., 2011). While beneficial effects of cognitive behavioral therapy and antidepressants have been reported for PSD (Starkstein & Hayhow, 2019; Wang et al., 2018), a Cochrane meta‐analysis concluded that effective interventions for PSF are lacking (Wu, Kutlubaev, et al., 2015), and more information about novel treatments is needed. A recent study on minimally impaired stroke patients reported reduction in fatigue after a single session of anodal transcranial direct current stimulation (tDCS) (De Doncker et al., 2021), a noninvasive brain stimulation technique using low‐amplitude direct currents to modulate cortical excitability. tDCS has also been applied to treat PSD (Valiengo et al., 2016; Valiengo et al., 2017), and associations between PSD and left dorsolateral prefrontal cortex (DLPFC) connectivity or damage (Egorova et al., 2017; Grajny et al., 2016) suggest that neuromodulative methods targeting this region may be particularly effective (Egorova et al., 2017). In line with this, the left DLPFC is a frequent target area for studies using anodal tDCS aiming to alleviate major depressive disorder (Bennabi & Haffen, 2018; Boggio et al., 2008), and tDCS applied to this area may thus have indirect positive effects on fatigue due to its assumed antidepressant effects. Additionally, an increasing number of studies have identified the DLPFC among the key regions associated with cognitive fatigue and motivation (Chong et al., 2017; Müller & Apps, 2019; Wylie et al., 2020), suggesting the possibility of direct positive fatigue effects from tDCS applied to this region as well. Yet, the evidence supporting beneficial effects of tDCS on PSD has been controversial (Bucur & Papagno, 2018), and the mechanisms of potential fatigue‐reducing effects remain elusive (De Doncker et al., 2021). Preliminary positive findings should thus be confirmed in larger and controlled studies.

Due to the assumed link between PSF and cognitive impairments, particularly within the domains of attention and processing speed (Johansson & Ronnback, 2014; Ulrichsen et al., 2020), cognitive rehabilitation was recently suggested as a potentially efficient treatment for alleviating fatigue (Aarnes et al., 2020). While significant improvements in fatigue after cognitive training have been reported in patients with multiple sclerosis (MS) (De Giglio et al., 2015), other studies revealed no significant effect on MS fatigue (Pérez‐Martín et al., 2017), and the feasibility of cognitive training for PSF has not been evaluated in chronic stroke patients. Further complicating matters, PSF and PSD may in itself impose considerable barriers to rehabilitation attendance and reduce the probability of positive outcomes (Chen et al., 2015; Llorca et al., 2015; Michael, 2002). Investigations of attendance and attrition rates and training gain in relation to fatigue may therefore provide important information for treatment choices.

In sum, effective treatment options for PSF are lacking, but preliminary evidence suggests that tDCS may have a potential to alleviate PSF and PSD. In a trial evaluating the clinical utility of combining cognitive training and tDCS to improve cognitive function post stroke (Kolskår et al., 2020; Richard et al., 2020), we quantified the effect of real stimulation versus sham on self‐reported fatigue and depression using Bayesian mixed‐effects models. Based on prior literature suggesting beneficial effects of tDCS on fatigue and depression in other conditions, we hypothesized that patients receiving real stimulation would display a larger reduction in symptoms compared to patients receiving sham stimulation. Due to the high comorbidity and symptom overlap between fatigue and depression, we examined the constituents of this relationship in further detail using an exploratory network approach to map symptom‐level centrality and associations at baseline and across time.

## 2 METHODS

### 2.1 Sample and study timeline

**FIGURE 1:**
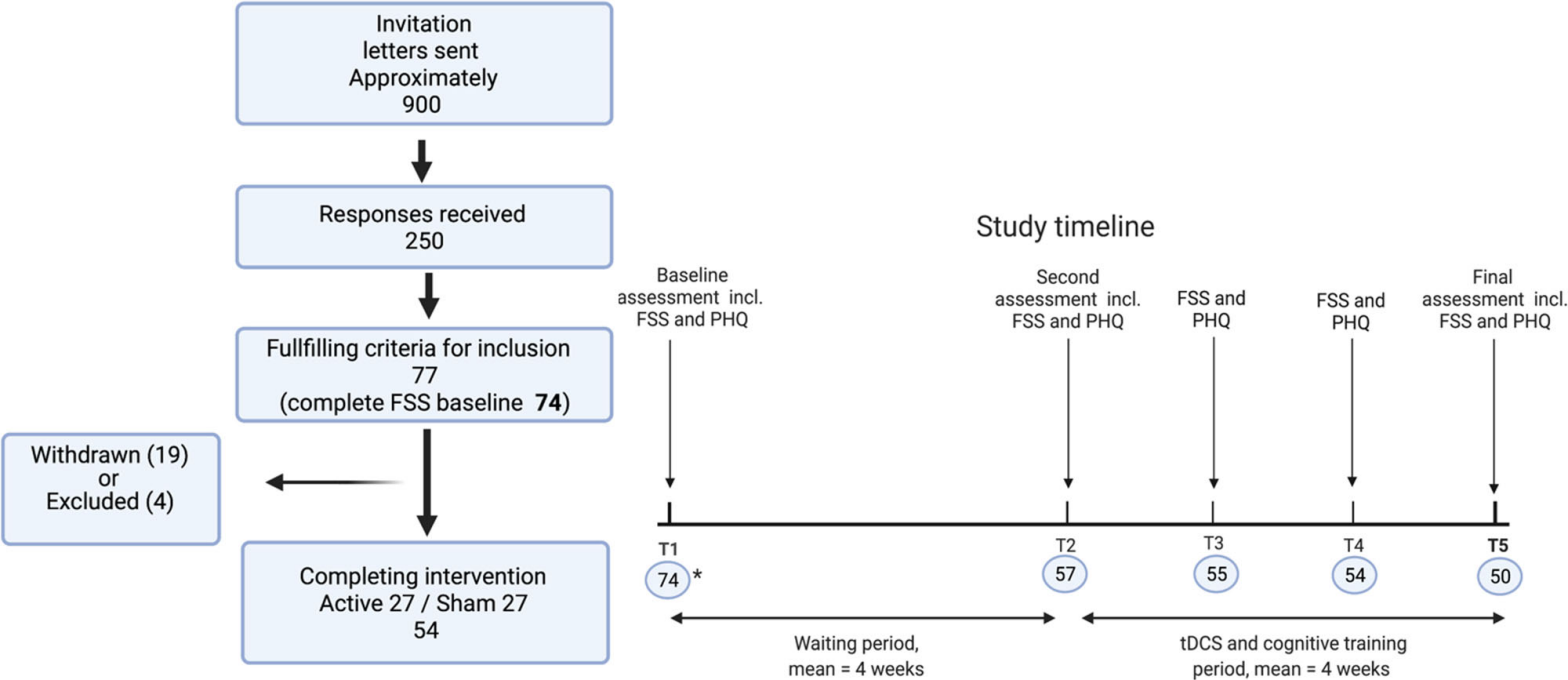
Flow diagram of recruitment (left) and study timeline (right). Number of patients with complete FSS scores is provided in blue circles

Figure 1 shows a schematic outline of the parallel group study timeline and a flow diagram of recruitment (see Kolskår et al. [2020] for a detailed description of overall study protocol). Stroke survivors in the chronic phase (>6 months from stroke onset) were invited to participate. All patients were previously admitted with acute stroke to the Stroke Unit, Oslo University Hospital, or the Geriatric Department, Diakonhjemmet Hospital. Exclusion criteria included severe neurological, neurodevelopmental, or psychiatric conditions prior to the stroke, MRI contraindications, and transient ischemic attack. All patients suffered mild to moderate strokes (National Institute of Health Stroke Scale [NIHSS; Meyer & Lyden, 2009] ≤7 at hospital discharge), and mean time from stroke onset was 26 months. We included patients with both ischemic and hemorrhagic strokes, but in the current sample, all but one patient suffered ischemic stroke. None of the included patients reported severe linguistic or visual impairments.

The study was approved by the Regional Committee for Medical and Health Research Ethics South‐East Norway (2014/694, 2015/1282). Participants provided written and informed consent prior to enrollment. All participants received a compensation of 500 NOK.

The majority (*n* = 14) of the 19 patients who withdrew from the study did so prior to the training, while five withdrew during the course of the intervention. None withdrew consent, therefore baseline data are reported on both completing and withdrawn patients. Three patients were excluded due to medical conditions occurring after inclusion, and one patient was excluded due to problems with fitting the MRI coil.

### 2.2 Cognitive training

Computerized working memory training was done using Cogmed QM (Cogmed Systems AB, Stockholm, Sweden). While there is a vast body of theoretical frameworks accounting for working memory, it is generally conceptualized as a system allowing the temporary storage and processing of information (Hitchcock & Westwell, 2017). Moreover, across different theoretical frameworks, working memory capacity is explicitly linked to attentional control (see, e.g., Engle, 2002; Engle et al., 1999; Kane et al., 2001), and Cogmed (2022) claims that working memory training improves sustained attention and cognitive control, increasing the ability to resist distraction. With consideration to recent work on fatigue, suggesting that PSF is associated with a deficit in filtering of distractors (Kuppuswamy et al., 2022, Preprint), Cogmed working memory training may have positive effects on fatigue through strengthening attentional control.

Details of implementation are described elsewhere (Kolskår et al., 2020; Richard et al., 2020). Patients completed 17 training sessions over 3–4 weeks, corresponding to approximately five weekly training sessions. Seven sessions were carried out at Oslo University Hospital, as patients simultaneously received tDCS or sham stimulation (see below) during six of these sessions. The remaining sessions were carried out at home. The Cogmed protocol included 12 different auditory–verbal and visual–spatial exercises, where each session consisted of eight exercises. Level of difficulty adjusts according to initial individual performance, and, to allow for calibration, we did not include the two first sessions for each task in the statistical analyses used to estimate performance gain. Tasks completed less than three times were also discarded. Training gain was calculated for the following eight exercises: Cube, Digits, Grid, Hidden Objects, Rotation, Sort, Twist, and 3D‐cube.

### 2.3 tDCS protocol

The tDCS protocol is described in detail by Kolskår et al. (2020). Briefly, an in‐house MATLAB script was used to randomize participants into either sham or active condition at study inclusion. A total of six tDCS sessions were administered, two times per week, and with a minimum of 48 h between each session. Active tDCS stimulation was administered at 1 mA, to minimize the risk of adverse effects. Each stimulation lasted for 20 min (ramp‐up time 120 s and fade‐out time 30 s). The sham stimulation was done by the fade‐in, short stimulation, fade‐out approach (Ambrus et al., 2012), with ramp‐up followed by 40 s of active stimulation before fade‐out in accordance with factory settings. Stimulation was delivered a by direct current stimulator (neuroConn DC stimulator plus, Germany), through 5 × 7 cm rubber pads covered with high‐conducting gel (Abralyt HiCl, Falk Minow Services Herrsching, Germany). The anodal electrode was placed over F3 (left DLPFC) and cathodal at O2 (right occipital/cerebellum in the 10–20 system).

### 2.4 Outcome measures

The present study reports on prespecified exploratory endpoints regarding fatigue and depression in a trial evaluating the clinical utility of combining cognitive training and tDCS to improve cognitive function post stroke (Kolskår et al., 2020; Richard et al., 2020).

#### 2.4.1 Fatigue and depression self‐report measures

Subjective fatigue was measured by the self‐report scale Fatigue Severity Scale (FSS) (Krupp et al., 1989), where impact of fatigue on different areas of daily life is rated from 1 to 7. FSS has demonstrated acceptable psychometric properties (Whitehead, 2009) and is frequently used to assess fatigue in neurological patient populations (Cumming et al., 2016). FSS scores are usually reported as mean values (lowest mean 1, highest mean 7), where higher scores indicate higher fatigue impact. The cutoff for clinically significant fatigue applied in the literature is either ≥4 (Nadarajah & Goh, 2015; Schepers et al., 2006) or ≥5 (Kjeverud et al., 2020; Lerdal et al., 2005; Morsund et al., 2019; Naess et al., 2012). A cutoff of ≥5 has been recommended to prevent overestimation of cases, as a cutoff of ≥4 resulted in 42% of healthy controls being identified as fatigued in a large (*N* = 1800) Norwegian sample (Lerdal et al., 2005). However, because both ≥4 and ≥5 are used in the literature, we here conduct analyses by both values for transparency.

Symptoms of depression were assessed by the depression module of the Patient Health Questionnaire (PHQ‐9) (Kroenke et al., 2001). PHQ is a nine‐item self‐report scale, where items correspond to the criteria of depression as stated in the Diagnostic and Statistical Manual of Mental Disorders (DSM‐IV) (American Psychiatric Association, 1994). The respondent indicates degree of symptom load on a scale ranging from 0 (*not at all*) to 3 (*nearly every day*), yielding a minimum total score of zero and a maximum score of 27, with ≥10 reflecting moderate depression (Kroenke et al., 2001).

Both fatigue impact and depression were assessed at five time points across an 8‐week period (see Figure 1 for timeline). The first assessment was collected approximately 4 weeks before the second assessment, and the four consecutive assessments were collected on a weekly basis.

#### 2.4.2 Supplementary cognitive measures at baseline

To test for associations between FSS, PHQ, and cognition at baseline, the following neuropsychological tests were included: Montreal Cognitive Assessment (MoCA; Nasreddine et al., 2005) and the subtests “Vocabulary” and “Matrix Reasoning” from Wechsler Abbreviated Scale of Intelligence, Second Edition (WASI‐II; Wechsler, 2011). In addition, the four‐trial version of the Stroop Color Word Interference test (CWIT) was applied to obtain a measure of cognitive speed, inhibition, and interference (Delis et al., 2001). The California Verbal Learning Test (CVLT‐II; Delis, 2000) was used as a measure of episodic verbal learning and memory. Here, we included the first trial and the total number of recalled words across five trials. The Cognitive Failures Questionnaire (CFQ) (Broadbent et al., 1982) was included as a subjective measure of memory, perception, and motor failures.

### 2.5 Statistical analyses

Statistical analyses were performed using R version 4.0.3 (R Core Team, 2020).

#### 2.5.1 Effects of tDCS and time on fatigue and depression

To assess effects of tDCS on fatigue and depression, and to quantify evidence in favor of the null and alternative hypothesis, we used Bayesian hypothesis testing. Mixed‐effect Bayesian regression models were created using the brms package (Bürkner, 2017) in the Stan computational framework (http://mc‐stan.org/). We estimated mixed models separately for FSS and PHQ, using FSS or PHQ as dependent variables. Time (1–5), tDCS group (sham, active), tDCS group × time, age, and sex were entered as fixed factors, and participant as random factor. The models were run using four chains with 8000 iterations each, in which the first 4000 iterations were discarded as burn‐in. Predictors were assigned normal priors with means of 0 and standard deviation of 1. All variables were standardized prior to analysis. As a means of ensuring that the inclusion of baseline time point 1 did not bias the results, we estimated follow‐up linear mixed‐effects models with and without baseline 1 included.

#### 2.5.2 Fatigue and cognitive training: Study withdrawal and training gain

Baseline group differences between patients who completed the intervention (*n* = 50) and patients who withdrew from the study (*n* = 19) were first examined by independent samples *t*‐tests. In a follow‐up analysis testing for specific effects of PSF (defined as mean ≥5/ ≥4 on FSS [Lerdal et al., 2005]) on study adherence, we estimated a logistic regression model with completing/withdrawing as outcome variable, and PSF status, PHQ scores, age, and sex as predictors.

To quantify individual Cogmed training gain, we followed the approach by Kolskår et al. (2020). Here, the effect of repeated training was calculated for each subtest by running linear models with task performance as dependent variable and session number as predictor variable for each participant, where the resulting beta‐estimates (slopes) reflect the change in performance across time/sessions. Multivariate outlier detection in terms of mean task performance and beta estimates was done by the mvoutliers package in R, using the aq.plot function (Filzmoser & Gschwandtner, 2018). Two of the subtests were discarded from further analyses due to a high number of outliers compared to the remaining tests (subtest “hidden objects” had eight identified bivariate outliers, subtest “digits” had seven, while the remaining subtests had zero or one).

We then tested for associations between fatigue and training gain by estimating linear models for each subtest, applying the beta estimate as dependent variable, and FSS score at TP1, age, and sex as independent variables. To assess whether potential effects were specific for fatigue, we re‐ran the same models with PHQ as predictor variable instead of FSS.

#### 2.5.3 Associations between FSS and baseline measures of cognitive performance

To test for associations between fatigue, depression, and cognitive function at baseline, we estimated linear models with task performance on neuropsychological tests as dependent variables, and baseline FSS score as independent variable, adding age, sex, and WASI scores as covariates in all models. In addition, we tested for an association between FSS and subjectively reported cognitive function, by estimating the same model with the Cognitive Failures Questionnaire (CFQ) as dependent variable.

#### 2.5.4 FSS and PHQ associations across time

##### Stability of specific symptoms

To get an estimate of stability and change in individual symptoms across time, we estimated the coefficient of variation (CV) for each FSS and PHQ item across time point 1–5, yielding one CV value per item for each person. As FSS and PHQ have different scale properties, the CV values between them cannot be compared directly, but CV estimates provide relevant information about relative stability or change in individual symptoms within each scale.

#### 2.5.5 Network estimations

We used the qgraph package in R (Epskamp et al., 2012) to estimate networks based on Spearman's rank order correlations matrices. We estimated two baseline networks (*n* = 74), one with sum FSS scores and individual PHQ items to investigate associations between overall fatigue severity and specific depressive symptoms, and one with individual FSS and PHQ items, to visualize item‐level associations. The first (sum FSS) network was estimated with regularized partial Spearman correlations via EBICglasso (tuning parameter set to 0.15), while the second (all‐item network) was based on full correlations due to the high numbers of parameters relative to sample size and associated stability issues caused by partial correlations. The latter procedure was repeated to estimate five all‐items temporal networks with completing patients only, and plotted according to principal component analysis (PCA) dimension loadings, allowing for comparison of network structure across time. PHQ item number 9, suicidal ideation, was excluded from network estimations due to an extremely positively skewed distribution of scores (mean = 0.08). We then estimated one individual‐item network based on full correlations for each time point (time point 1–5) to investigate item centrality across time. Here, only completing patients (*n* = 50) were included.

#### 2.5.6 Node centrality and stability

The relative importance of a node (item) in the network can be evaluated by various centrality measures. We estimated strength centrality, which is a stable (Fried et al., 2016) and commonly examined centrality measure in psychological networks (Malgaroli et al., 2021), representing the sum of all absolute edge weights directly connected to a given node (Bringmann et al., 2019). To evaluate the relative importance of symptoms across time and networks, we followed the approach by Malgaroli et al. (2021), estimating one network per time point (1–5), and ranked node strength centrality for each time point, before calculating mean across‐time centrality ranking for each item. To investigate whether item centrality was associated with symptom severity, we calculated Spearman correlations between mean ranked item centrality and mean item scores. Stability was assessed using case‐dropping bootstrap (nBoots = 1000) by the bootnet package (Epskamp et al., 2018), where network models are estimated on subsets of the data. The correlation stability coefficient (CS) represents the maximum proportion of cases in the sample that can be dropped, maintaining a 95% probability that the correlation between the original centrality scores and the subsets’ centrality scores is minimum 0.70. A commonly applied rule of thumb is that the CV coefficient should not be lower than 0.25, while a coefficient above 0.50 indicates a relatively stable network (Epskamp et al., 2018). Edge accuracy and robustness were tested by bootstrapped estimations of edge confidence intervals and difference test for edges (Epskamp et al., 2018).

## 3 RESULTS

### 3.1 Effect of tDCS and time on fatigue and depression

**FIGURE 2:**
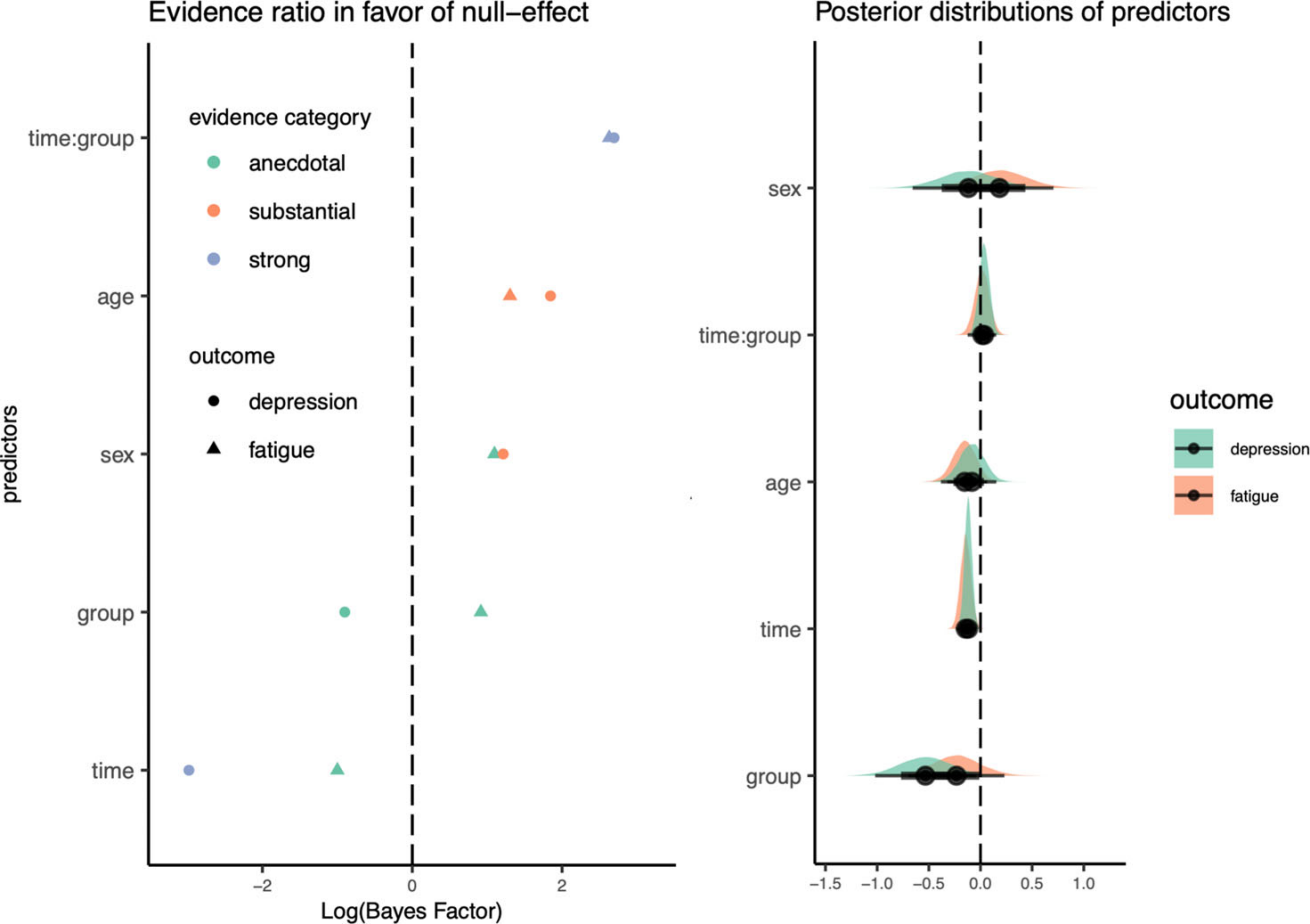
Estimated evidence ratio (left) and posterior distributions of predictors (right). Log(BF) > 0 represent evidence in favor of the null hypothesis and log(BF) < 0 represent evidence in favor of the alternative hypothesis. Posterior distributions of predictors (right) for the fatigue model (red) and depression (green) model

Figure 2 (left) shows log‐transformed Bayes Factor evidence plotted for the null hypothesis (intervention has no effect on FSS and/or PHQ) and alternative hypothesis (intervention has effect on FSS and/or PHQ) for all included predictors in the fatigue and depression models. Figure 2 (right) shows the posterior distributions of the coefficients of the standardized predictors for the fatigue model and depression model. Both models provided strong evidence (BF_01_ > 10, log‐transformed BF_01_ > 2) for the null hypothesis of no tDCS treatment effect (no time by group interaction effect on fatigue or depression) relative to the alternative hypothesis. Results also provided strong evidence for decreasing symptoms of depression over time (BF_01_ = 0.05, log‐transformed BF_01_ < −2), and anecdotal to moderate evidence for reduced fatigue over time (BF_01_ = 0.36, log‐transformed BF_01_ = −1). Follow‐up linear mixed‐effects models estimated without baseline 1 included revealed similar results regarding both the effect of tDCS and main effects of time.

**FIGURE 3:**
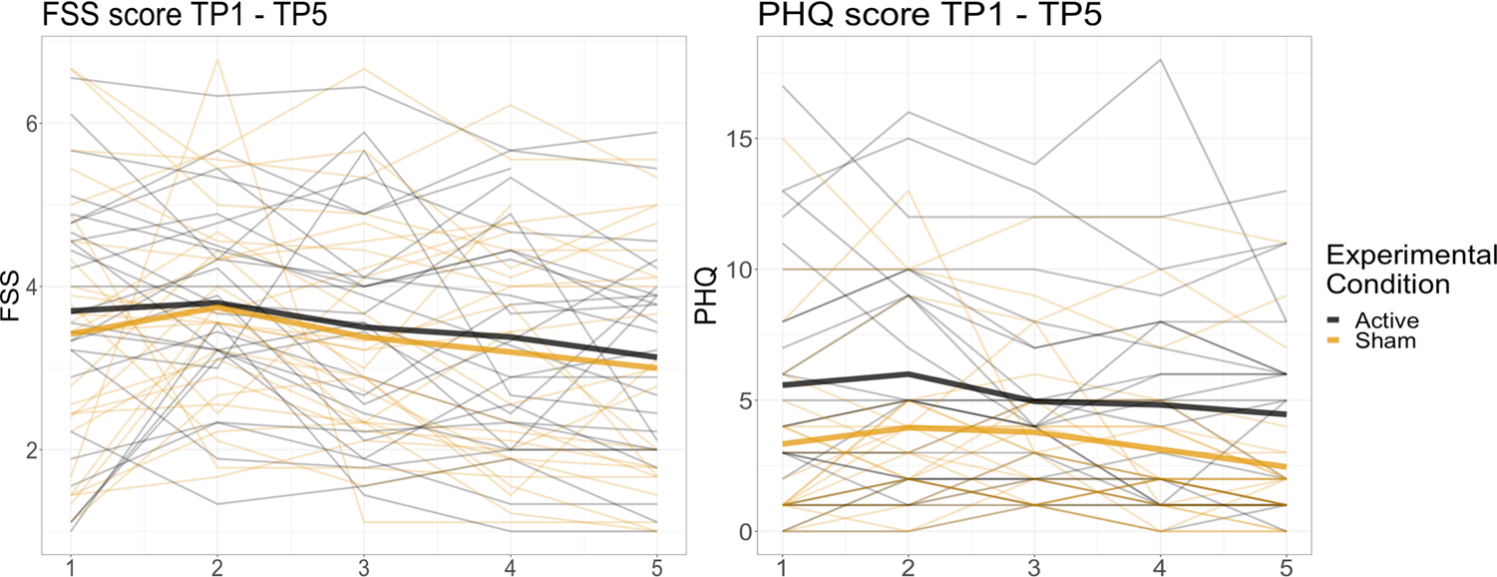
Individual FSS and PHQ scores across time. Scores are grouped by experimental condition (active vs. sham)

Figure 3 shows individual FSS and PHQ scores plotted by group for each time point (1–5). FSS mean score for completing patients (*N* = 50) at baseline was 3.5 (*SD* = 1.5), and 3.0 at TP5 (*SD* = 1.3). Corresponding sum PHQ values were 4.3 at baseline (*SD* = 4.2) and 3.5 (*SD* = 3.3) at TP5.

Follow‐up analyses revealed no evidence for a correlation between baseline FSS scores and individual slopes (BF_01_ = 1.33, log‐transformed BF_01 _= 0.12), but provided strong evidence for a correlation between baseline PHQ and individual slopes (BF_01_ = 0.001, log‐transformed BF_01 _< −2), suggesting that higher baseline PHQ scores were associated with a larger reduction in PHQ symptoms over time

### 3.2 Fatigue and computerized cognitive training: Study withdrawal and training gain

Descriptive information on patients who completed the study and patients who withdrew during the study is reported in Table 1. Group differences in continuous variables were tested by independent samples *t*‐tests, and we reported both *t*(*p*) values and Bayes Factors_10_, while group differences in sex were tested by Chi‐square test of independence.

**TABLE 1:** Group differences between withdrawn and completing patients

	Withdrawn (*n* = 19)	Completing (*n* = 54)	Difference withdrawn and completing patients	
	Mean (*SD*)	Mean (*SD*)	*t*(*p*)	BF_10_
Age	60.9 (17.1)	69.1 (7.3)	2.05 (.051)	**7.7**
Sex (*χ* ^2^)	10 M/9 F	40 M/14F	2.99 (.083)	
Education	14.3 (3.9)	14 (3.7)	0.38 (.702)	0.30
NIHSS	1.3 (1.6)	1.3 (1.5)	0.04 (.96)	0.28
Months since stroke	29 (7.7)	25 (9.1)	−1.70 (.093)	0.70
Lesion volume^a^	9239 (15,459)	5978 (9616)	−0.79 (.435)	0.30
FSS (TP1)	4.6 (1.5)	3.5 (1.5)	−2.41 (.022)*	**5.86**
PHQ (TP1)	5.9 (4.2)	4.3 (4.6)	−1.25 (.190)	0.69
GAD	3.2 (2.7)	2.54 (3.5)	−0.74 (.461)	0.35
MoCA	24.7 (4.0)	25.9 (2.7)	1.13 (.268)	0.59
IQ	110 (16.5)	110 (16.9)	0.05 (.954)	0.30

^a^ One (completing) patient constituted an extreme outlier in terms stroke volume (∼8 *SD*s above the mean) and was removed from the group difference test of lesion volume.

* Signifies p‐values < .05. Bold values signify moderate (BF_10_ > 3) evidence for group differences.

Independent samples *t*‐tests revealed significantly higher fatigue and lower age in patients who withdrew from the study, than in patients who completed the intervention. Follow‐up logistic regression analyses including fatigue status (mean score ≥5 or ≥4 on FSS), PHQ scores, age, and sex as predictors for completing/withdrawing from the study mirrored results from the *t*‐tests. Patients with severe fatigue (mean score ≥5 on FSS) were considerably more likely to withdraw from the study compared to patients without fatigue (odds ratio [OR] = 1.83, 95% confidence interval [CI]: 0.27 to 3.52, *p* = .024), but when including patients with moderate fatigue (mean score ≥4 on FSS), only age was a significant predictor for withdrawal (OR = 0.05, 95% CI: −0.00 to 0.11, *p* = .034).

Linear regression models revealed a significant, negative association between FSS and Cogmed beta slopes in three of the six included training tasks (3D Cube: *b* = −0.01, *t*(48) = −2.87, *p* = .006 [FDR‐corrected *p *= .021]; Grid: *b* = −0.01, *t*(48) = −2.81, *p* = .007 [FDR‐corrected *p* = .021]; and Sort: *b* = −0.01, *t*(48) = −2.09, *p* = .042 [FDR‐corrected *p* = .094]). However, the effects were small with considerable variation around the regression line, reflected in adjusted *R* ^2^ values of .07 and .09. Of note, the associations between FSS and Cogmed beta slopes were negative in all models, but only reached significance for three out of six training tasks. The analyses did not provide evidence for any association between training gain and age, sex, or IQ. For illustration purposes, Cogmed slopes are plotted against FSS and PHQ in Figure S1. Results from regression models estimated with PHQ as independent variable instead of FSS mirrored the fatigue models, with a significant negative association between PHQ and training slopes identified in two of the same tasks (3D Cube: *b* = −0.004, *t*(48) = −3.92, *p* < .001 [FDR‐corrected *p* = .003]; Grid: *b* = –0.001, *t*(48) = −3.21, *p* = .002 [FDR‐corrected *p* = .014]). Depression models explained slightly more variance, with *R* ^2^ values of .18 and .12, respectively.

There were no evidence supporting an association between FSS scores and performance on baseline neuropsychological tests (Table S2). However, FSS was significantly associated with subjectively reported cognitive failures.

### 3.3 FSS and PHQ associations by baseline and across‐time networks

**FIGURE 4:**
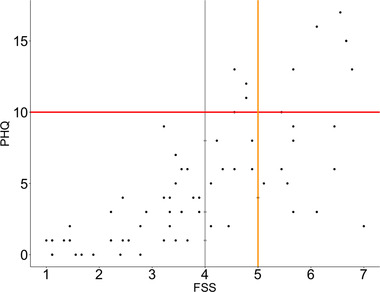
Individual mean scores of FSS (x‐axis) and PHQ sum scores (y‐axis) plotted for all patients (n = 74). Vertical lines (gray and orange) mark commonly used cutoff values for clinical fatigue, while horizontal red line marks cutoff value for depression

Figure 4 depicts individual mean FSS scores at baseline plotted against sum PHQ. Bayes factor estimations for linear correlations provided strong evidence for a positive association between the measures (BF_10_ > 150, mean posterior = 0.68, 97.5% CI = [0.55–0.79]).

All patients scoring above clinical cutoff for moderate depression on PHQ also experienced moderate or severe fatigue. The association was not reciprocal, in that several patients reported high FSS scores, without displaying clinical levels of depression. Table S1 shows the individual items in FSS and PHQ, along with the mean aggregated scores (TP1–TP5), standard deviations, and CV values, indicating degree of variability across time points.

**FIGURE 5:**
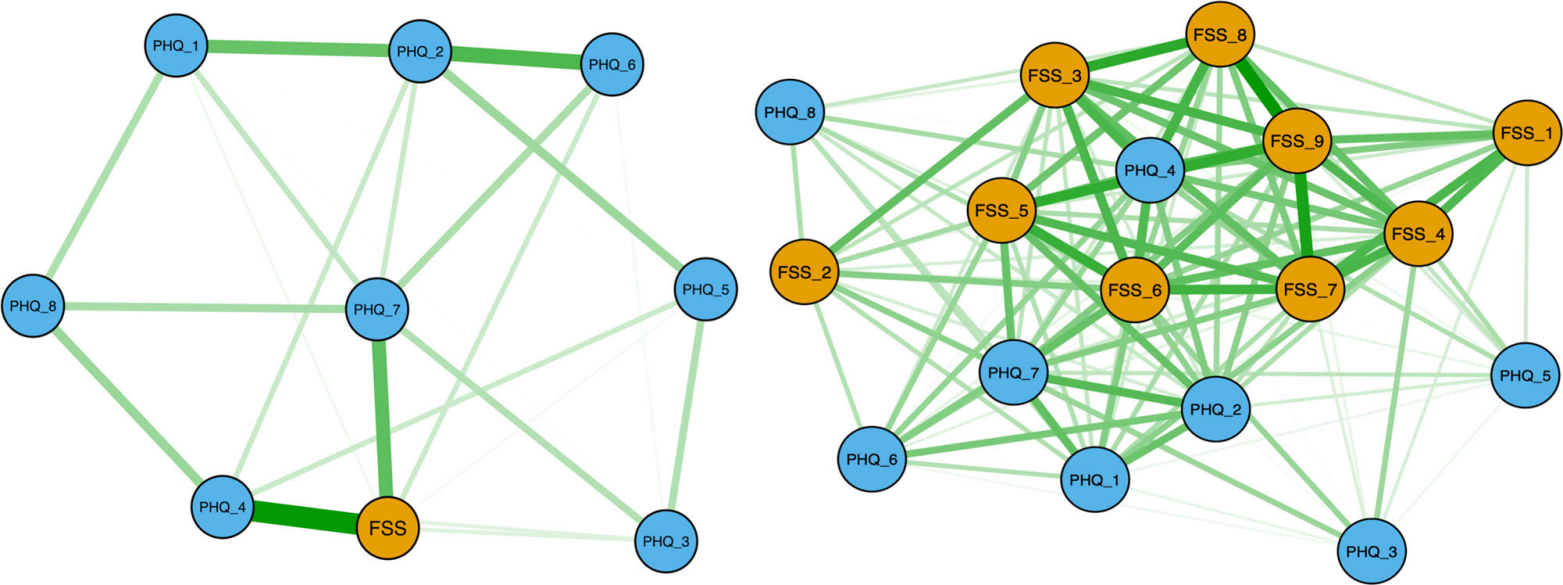
Associations between baseline FSS and PHQ. Network visualization of Spearman partial correlations with EBICglasso regularization (tuning parameter = 0.15), between FSS sum score and PHQ items for all patients (*n* = 74) at baseline (left). Network visualization of full Spearman correlations between all FSS and PHQ items at baseline (right). Green edges signify positive correlations, while red edges (none present) represent negative correlations (Epskamp et al., 2012). The thickness of the lines indicates the strength of the association

Figure 5 shows baseline networks estimated with FSS sum scores and individual PHQ items (left) and all FSS and PHQ items (right). The sum FSS graph indicated that PHQ items reflecting tiredness, lack of energy, and trouble concentrating showed strongest associations with overall fatigue. However, only one edge “sum FSS – PHQ 4 (tiredness/lack of energy)” was identified as significantly different from the majority of other network edges by bootstrapped difference test for edge‐weights (Figure S2). Item strength centrality (CS‐coefficient) for network 1 (sum FSS and PHQ scores) at baseline was estimated to be .28 by case‐dropping bootstrap sampling, meaning that up to 28% of the sample could be dropped while retaining a correlation of .70 with the original sample strength centrality (95% CI). Corresponding CS‐coefficient for baseline network based on full correlations between all items (right) was estimated to be .51. Bootstrapped difference test for edge‐weights revealed that most edges were not significantly different from the majority of other edges (Figure S3), with the strongest edge being FSS 8 (“Fatigue is among most disabling symptoms”) and FSS 9 (“Fatigue interferes with my work, family, or social life”). Bootstrapped CIs (Figure S4) showed a substantial overlap between edge‐weights, indicating that order of edges should be interpreted with caution (Epskamp et al., 2018).

Temporal networks based on full correlations are shown in Figure S5 and corresponding plots for bootstrapped difference test for node strength in Figure S6. Nodes are placed according to loadings on unrotated PCA dimensions. While this approach should be considered exploratory due to the high number of items compared to sample size, the temporal network graphs show that associations between symptoms/network structure vary across times of measurement, and suggest that fatigue and depression items tend to cluster according to their respective scales.

**FIGURE 6:**
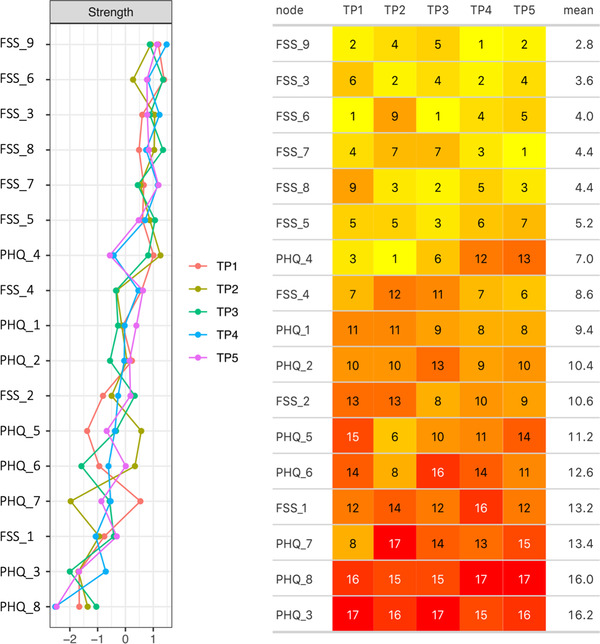
Item strength centrality across time points. Standardized strength node centrality of the 17 FSS/PHQ items across five time points (left), and heatmap table (right) showing the ranked node strength centrality of the five networks, estimated at time point 1–5. Each node is ranked in decreasing order, from 1 (highest centrality) to 17 (lowest centrality). FSS item number 9 (“Fatigue interferes with my work, family, or social life”) demonstrated the highest mean ranked strength centrality across time, followed by FSS item 3 (“I am easily fatigued”)

Figure 6 shows estimated standardized strength centrality (left) and ranked centrality (right) for individual items at each time of measurement, suggesting reasonable consistency in strength centrality across time for most items, except PHQ item 7 (concentration problems), 5 (appetite), 4 (tired/little energy), and 6 (feeling bad about yourself). Evidence for an association between overall symptom severity (calculated as mean item score across time 1–5) and overall item centrality (estimated as mean ranked centrality across time) was moderate (BF_10_ = 3.1, mean posterior = −0.40, 97.5% CI = [−0.71 to 0.03]) with wide credible intervals, indicating that higher symptom severity is associated with increased symptom centrality, but that the strength of the relationship is uncertain.

## 4 DISCUSSION

In a sample of chronic stroke patients, we tested the add‐on effect of tDCS combined with computerized cognitive training on fatigue and depressive symptoms. While symptoms of fatigue and depression decreased over the course of the intervention, Bayesian analyses provided strong evidence supporting no added beneficial effect of tDCS on fatigue or depression severity. To our knowledge, no prior studies have examined *longitudinal* effects of tDCS on PSF in a randomized controlled trial, although a recent study reported reduced FSS scores in stroke patients after a single session of anodal tDCS applied bilaterally to the primary motor cortex (De Doncker et al., 2021). Direct comparison between studies is complicated due to differences in protocols, regarding both electrode montage and location (both in terms of the cathode and anode), stimulation frequency, number of sessions, and current amperage. There might be greater treatment benefit with higher number of sessions and increased stimulation intensity (Charvet et al., 2018). Although comprehensive evidence for a linear relationship between intensity and response is still lacking (Esmaeilpour et al., 2018), we cannot rule out the possibility that we would have observed stronger effects of treatments using a higher stimulation intensity. Moreover, tDCS treatment response may interact with individual characteristics such as time since stroke onset, lesion location, or lesion size. For example, while Saiote et al. (2014) found no group effects of tDCS on subjective fatigue in patients with MS, a correlation was reported between lesion load in left frontal cortex and treatment response. Future well‐powered studies including patients sampled from a wide severity spectrum may be able to discern associations between treatment response and clinical stroke and lesion characteristics.

The absence of tDCS effects may also be related to the cognitive training itself. fMRI studies have reported that fatigued patients display increased activation (Kohl et al., 2009) or impaired deactivation (Berginström et al., 2018) while performing cognitive tasks. Such aberrant network activation has been suggested to reflect suboptimal processing efficiency or increased cerebral “effort,” which in turn might manifest as subjective fatigue. Following this line of interpretation, the cognitive training may itself have evoked sensations of fatigue, and thus counteracted the effects of tDCS. On a related note, tDCS‐induced plasticity is dependent on the state (passive vs. performing cognitive/motor tasks) of the subject (Antal et al., 2007), implying that we cannot establish whether tDCS administered without simultaneous cognitive training could generate different results.

Nonrandom attrition is a frequent challenge in clinical intervention studies. Our attrition rate of approximately 26% insinuates that the demands of study participation were unacceptable to a fair proportion of the included patients. On average, the patients who completed the intervention were older and reported lower fatigue scores at baseline compared to the patients who withdrew. We found no group differences in baseline performance on neuropsychological tests, possibly indicating that fatigue constitutes a larger barrier to treatment adherence than cognitive impairments in mildly impaired stroke patients. Importantly, most patients withdrew prior to, and not during the cognitive training, implying that there is no strong basis to infer that the intervention regime was intolerable to patients with fatigue. Rather, one may speculate that patients with high fatigue considered the scope of the intervention in terms of both testing and training to be too demanding, and thus chose to withdraw at an early stage. Moreover, the lower age in patients who withdrew from the study may be explained in part by presumably higher family and work obligations among the younger population, conflicting with the time and labor intensity of the intervention.

While the current results provided no evidence of an association between symptoms of fatigue or depression and baseline neuropsychological test performance, both FSS and PHQ were negatively associated with training gain in two and three of six included subtests, respectively. Although effects were small, this mirrors results from our previous findings of no baseline cognitive associations with PSF, but evidence of declining performance during a task requiring sustained mental effort (Ulrichsen et al., 2020). While Ulrichsen et al. (2020) examined fatigue effects during a 20‐min attentional response time task, we here extend this finding to the beneficial effects of an intervention spanning several weeks. In contrast to our previous study, the current negative association with training gain was not specific to fatigue, as similar, slightly stronger, association was found for depression. This discrepancy may be due to the difference in performance measures, where the current study targeted change in performance in a range of complex tests over several weeks, reflecting learning rate/gain from repeated practice, while Ulrichsen et al. (2020) measured change in response times in a simple, attentional task at a single session, thus corresponding more closely to the concept of fatiguability (Kluger et al., 2013).

The time‐resolved network analyses suggested overall higher strength centrality of fatigue items (except item 1 and 2) than depression items. The central role of fatigue echoes a recent network meta‐analysis on depressive symptoms, identifying fatigue as the symptom displaying highest strength centrality across studies (Malgaroli et al., 2021). While the design of the current study does not allow for causal inference, it has been speculated that fatigue exacerbates depression after stroke (Ormstad & Eilertsen, 2015), suggesting that the risk of depression can be reduced with adequate management of fatigue. Following this line of interpretation, the finding that FSS item number 9 (“fatigue interferes with my work, family, or social life”) displayed the highest strength centrality while simultaneously being among the most stable FSS items across time may indicate that fatigue inhibiting social and professional obligations is particularly stressful and predisposes for worsening of respective symptoms. Yet, this hypothesis is based on cross‐sectional analyses, and its relevance should be tested in future longitudinal studies disentangling the causal relationship between individual symptoms.

Of note, the two FSS items displaying lowest ranked node strength centrality at baseline and across time were item 1 (“my motivation is lower when I am fatigued”) and item 2 (“exercise brings on my fatigue”). These specific items further demonstrated the lowest (item 1) and highest (item 2) CV values across time, compared to other FSS items. This accords with a previous report of poor psychometric properties for item 1 and 2 (Lerdal et al., 2005) and improved potential to detect fatigue changes across time after removal of these items (Lerdal & Kottorp, 2011).

The results should be interpreted considering several limitations. First, the patients suffered from relatively mild strokes, as reflected in the low NIHSS scores, possibly compromising generalizability of results to more severe patient samples. Also, although both fatigue and depressive complaints were comparable to previous chronic phase stroke studies (Cumming et al., 2018; Dajpratham et al., 2020; Valko et al., 2008), and a substantial proportion reported symptoms above clinical thresholds, we cannot rule out that we would have observed stronger effects of treatment in a sample with higher symptom load. Second, fatigue was measured by FSS, which provides a rather coarse measure of a multifaceted phenomenon. While FSS is a widely applied and validated measure, it is less sensitive to specific aspects of fatigue, for example, mental fatigue. It is thus conceivable that a more detailed measure enabling differentiation between fatigue subtypes could reveal effects or associations not detected by FSS. Third, as the final assessment of fatigue and depression was collected shortly after the last tDCS stimulation, the current study does not capture potential long‐term effects of stimulation, as identified in previous studies (Ayache et al., 2017; Li et al., 2019). Future studies should aim to evaluate long‐term effects in addition to immediate response.

Lastly, due to the lack of control group for the cognitive training, we cannot establish whether the observed reduction in fatigue and depressive symptoms was caused by the training or by other unknown factors such as anticipation, positive effects of interacting with the research staff, or statistical phenomena such as regression to the mean.

In conclusion, the current study investigated whether tDCS combined with computerized cognitive training alleviated symptoms of fatigue and depression in a sample of chronic phase stroke patients. Compared to sham stimulation, Bayesian analyses provided strong evidence of no additional effect of tDCS. Follow‐up analyses of attrition rate, individual differences in training gain, and item‐level network analyses of fatigue and depression scales support the notion of fatigue as a central clinical symptom, with possible implications for treatment adherence and response.

## AUTHOR CONTRIBUTIONS

K.M.U. and L.T.W. conceptualized the idea of the study. K.M.U., K.K.K., G.R., E.S., and A.M.S. curated the data. K.M.U., M.L.P., and L.M. performed formal analyses. K.M.U. and M.L.P. created figures and tables. K.M.U. wrote the original draft. K.K.K., G.R., and D.A. designed experiments. S.T. and L.T.W. performed supervision. L.M., A.E., and H.I.‐H. interpreted the results. J.E.N. and L.T.W. administered the project and acquired funding. All authors reviewed and edited the manuscript.

## CONFLICT OF INTEREST

The authors declare no conflict of interest.

### PEER REVIEW

The peer review history for this article is available at https://publons.com/publon/10.1002/brb3.2643.

## Supporting information

Supporting Information

## Data Availability

The data that support the findings of this study are available from the corresponding author upon reasonable request.
