# Supplementary material for: No add‐on effect of tDCS on fatigue and depression in chronic stroke patients: A randomized sham‐controlled trial combining tDCS with computerized cognitive training
Source: Brain Behav. 2022 Jun 6;12(7):e2643. doi: 10.1002/brb3.2643 (PMC9304833; doi:10.1002/brb3.2643)
Supplement: Supplementary file 1 — Supporting Information [file BRB3-12-e2643-s001.pdf]

Supplementary material

Supplementary Figure 1. Baseline FSS and PHQ scores plotted against Cogmed slopes

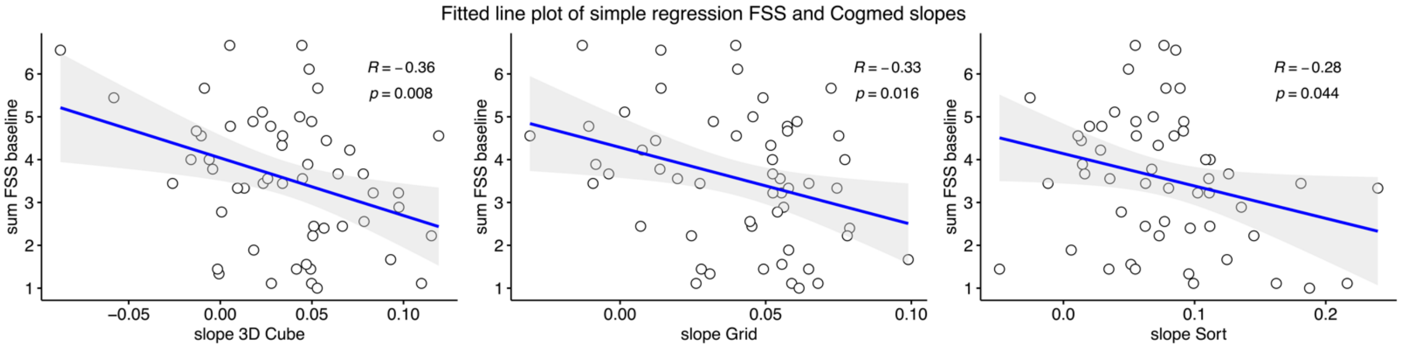

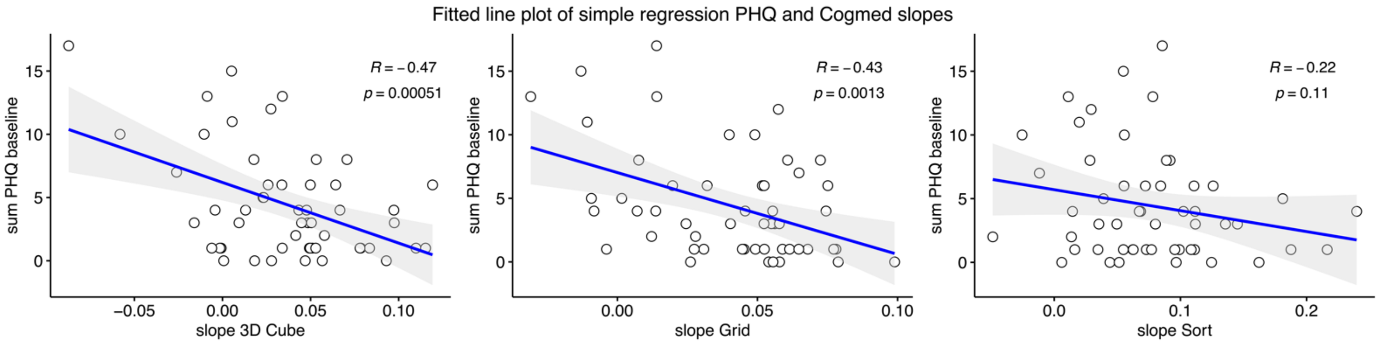

Supplementary Table 1. Mean aggregated scores (TP1 – TP5) and CV values for FSS and PHQ items.

	Fatigue Severity Scale<br>Score range 1 - 7	Mean	SD	CV
1	My motivation is lower when I am fatigued	22.5	7.4	31
2	Exercise brings on my fatigue	14.3	6.9	39
3	I am easily fatigued	15.4	6.9	36
4	Fatigue interferes with my physical functioning	19.5	6.7	33
5	Fatigue causes frequent problems for me	13.1	6.0	37
6	My fatigue prevents sustained physical functioning	15.7	7.3	36
7	Fatigue interferes with carrying out certain duties and responsibilities	16.7	7.0	35
8	Fatigue is among my most disabling symptoms	16.3	8.0	34
9	Fatigue interferes with my work, family, or social life	15.1	7.0	32

	Patient Health Questionnaire (PHQ – 9)<br>Score range 0 -3	Mean	SD	CV
1	Little interest or pleasure in doing things	2.4	2.6	20
2	Feeling down, depressed, or hopeless	6.3	5.6	16
3	Trouble falling or staying asleep, or sleeping too much	8.0	6.3	23
4	Feeling tired or having little energy	7.8	6.2	22
5	Poor appetite or overeating	5.2	5.4	14
6	Feeling bad about yourself — or that you are a failure or have let yourself or your family down	2.1	2.3	19
7	Trouble concentrating on things, such as reading the newspaper or watching television	1.9	2.4	17
8	Moving or speaking so slowly that other people could have noticed? Or the opposite — being so fidgety or restless that you have been moving around a lot more than usual	1.0	1.5	12
9	Thoughts that you would be better off dead or of hurting yourself in some way	0.3	1.0	4

*CV = coefficient of variation. We estimated one across-time CV value per item for each patient, and CV value in the table is the *mean* of these individual CV values.

Supplementary Figure 2. Bootstrapped difference test for edges (left) and node strength centrality (right), for baseline network estimated with FSS sum score and all PHQ items.

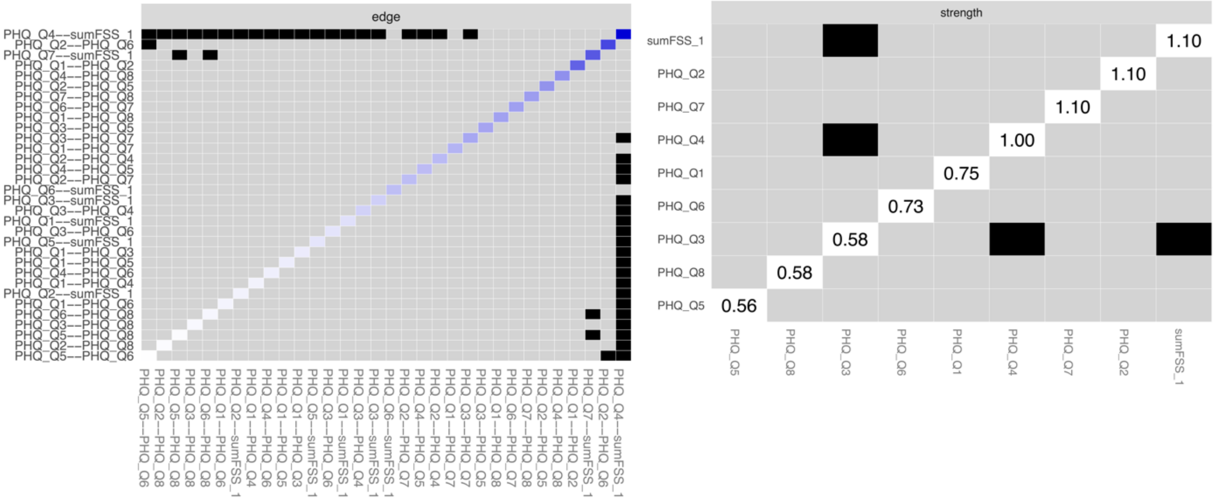

Supplementary Figure 3. Bootstrapped difference test for edges in full (all-item) baseline network.

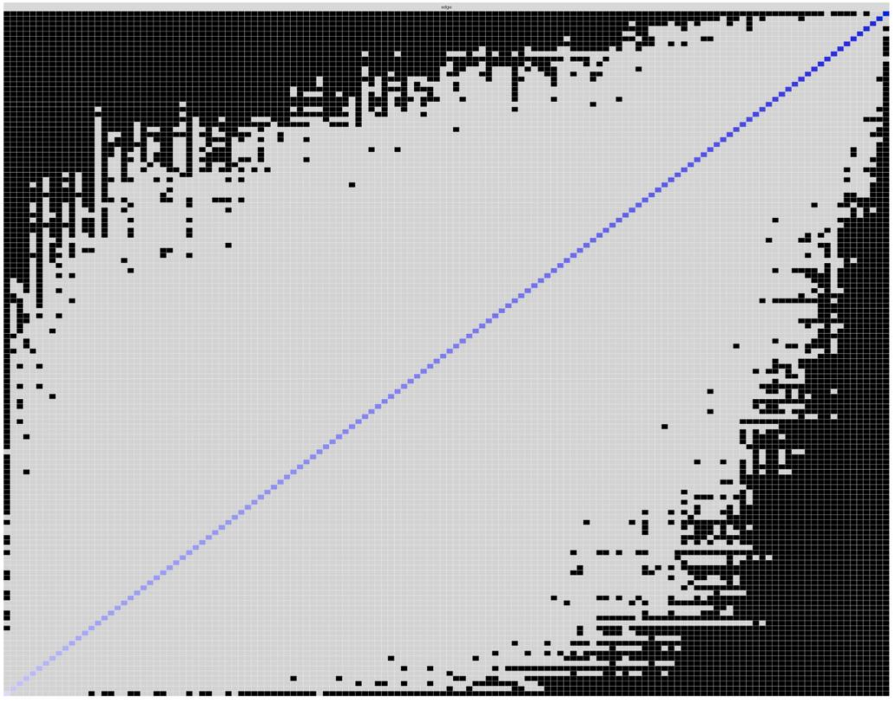

Bootstrapped edge difference tests ($\alpha = 0.05$) between non-zero edge-weights (153 in total) in the estimated network from baseline. Names have been removed due to abundancy. Grey boxes indicate that edges do not differ significantly from other edges.

Supplementary Figure 4. Bootstrapped CIs around edge-weights

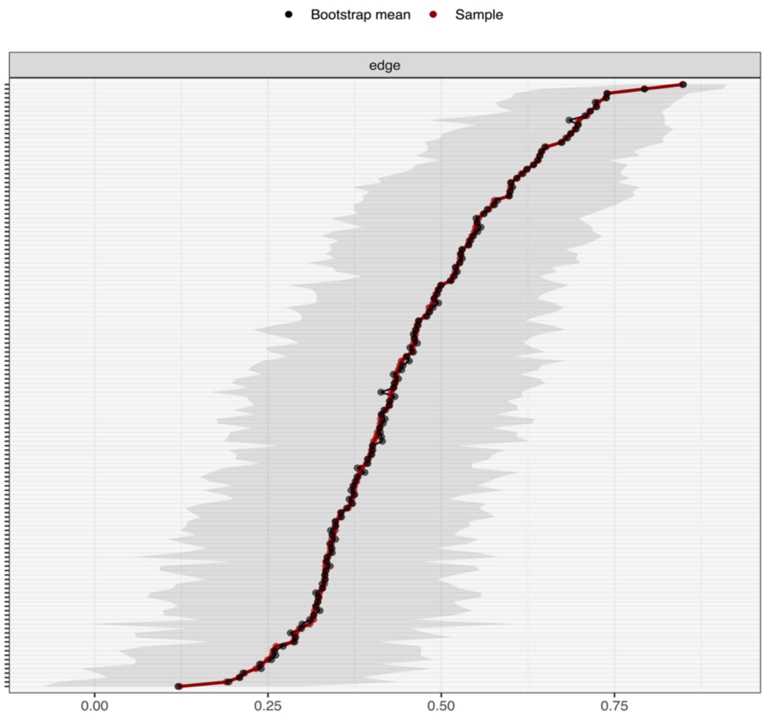

Figure 4. Bootstrapped CIs (grey area) around estimated edge-weights in the baseline network. Each line on the y-axis represent an edge (names have been removed due to abundancy). Most CIs are of considerable size and overlap with other edge-weights and suggests that interpretation of edge order should be done with caution.

Supplementary Figure 5. Unregularized, individual item networks of FSS and PHQ scores for each time point. Node placement is defined by loadings on (unrotated) PCA dimensions.

TP1

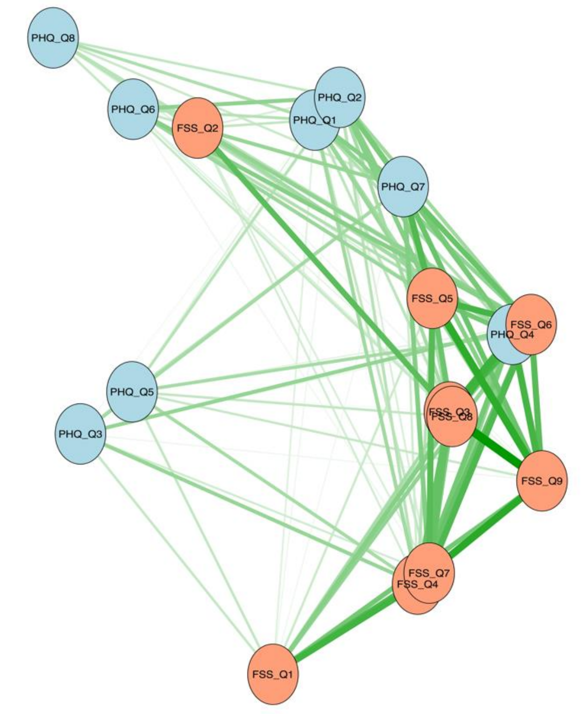

TP2

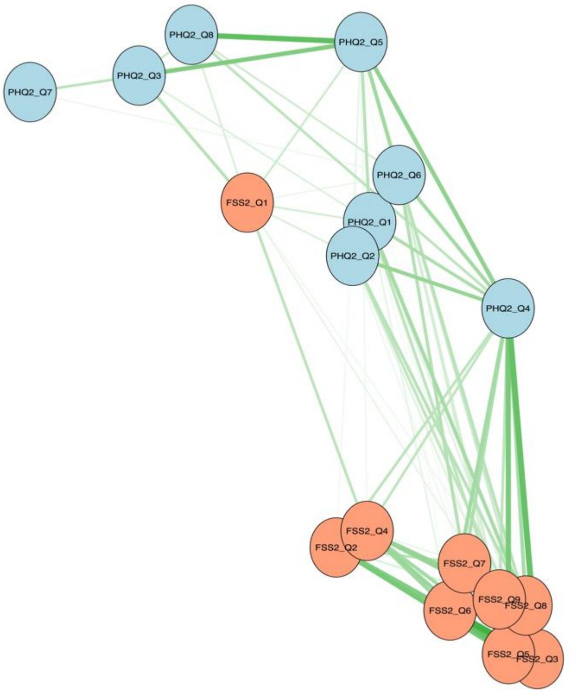

TP3

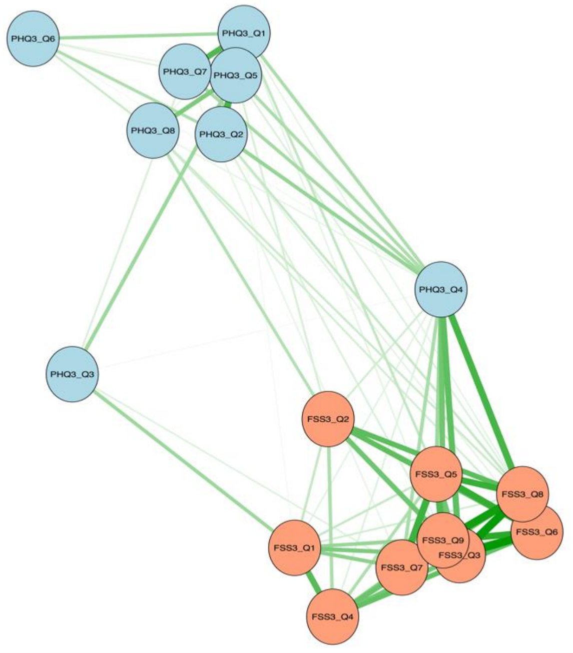

TP4

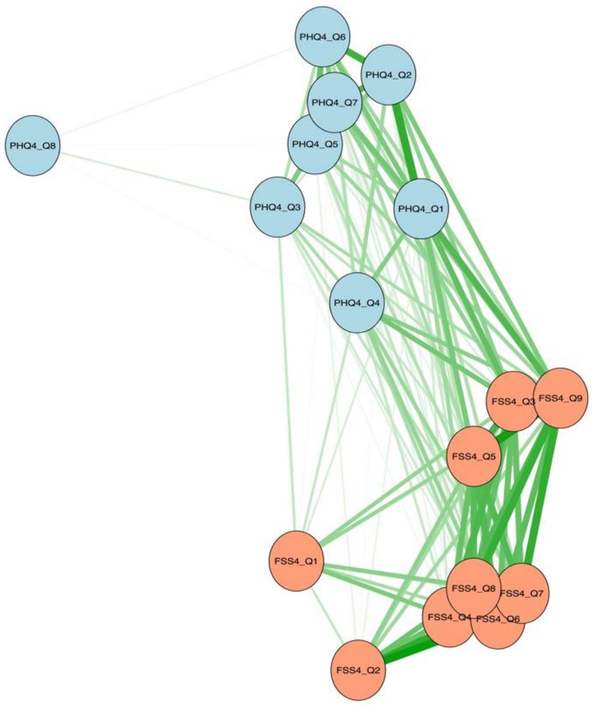

TP5

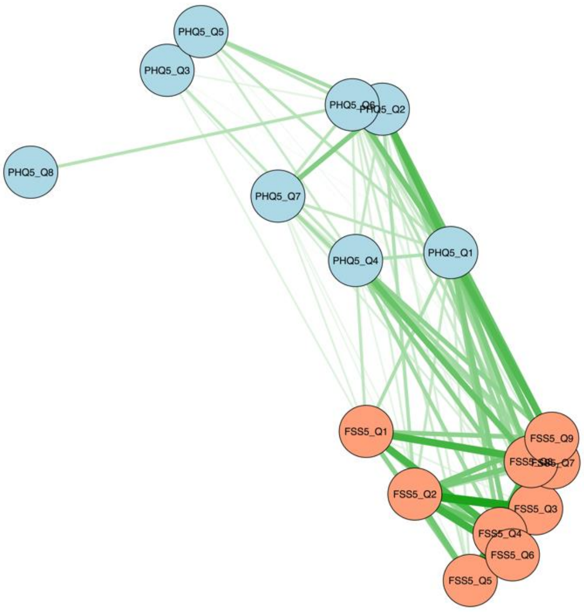

Supplementary Figure 6. Bootstrapped difference tests for node strength centrality in all-item networks, time-point 1 to 5.

TP1

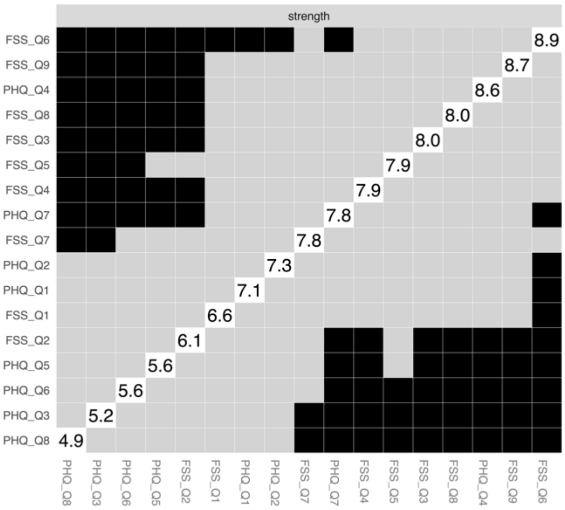

TP2

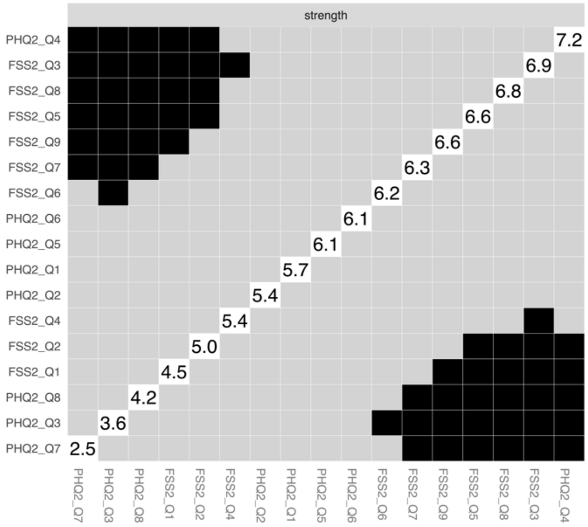

TP3

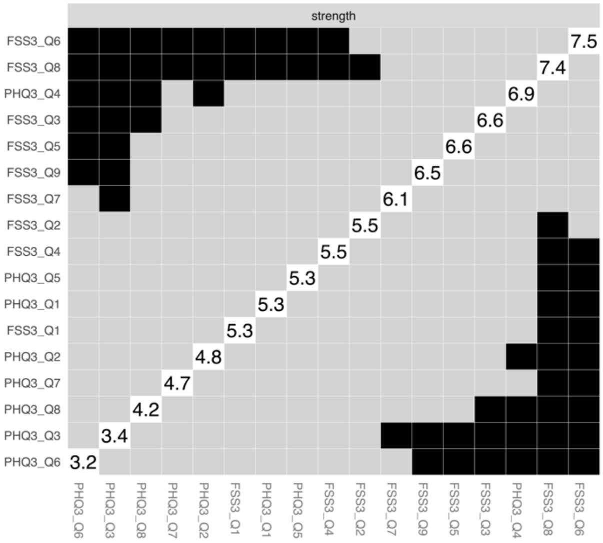

strength
FSS3_Q6
FSS3_Q8
PHQ3_Q4
FSS3_Q3
FSS3_Q5
FSS3_Q9
FSS3_Q7
FSS3_Q2
FSS3_Q4
PHQ3_Q5
PHQ3_Q1
FSS3_Q1
PHQ3_Q2
PHQ3_Q7
PHQ3_Q8
PHQ3_Q3
PHQ3_Q6
7.5
7.4
6.9
6.6
6.6
6.5
6.1
5.5
5.5
5.3
5.3
5.3
4.8
4.7
4.2
3.4
3.2
PHQ3_Q6
PHQ3_Q3
PHQ3_Q8
PHQ3_Q7
PHQ3_Q2
FSS3_Q1
PHQ3_Q1
PHQ3_Q5
FSS3_Q4
FSS3_Q2
FSS3_Q7
FSS3_Q9
FSS3_Q5
FSS3_Q3
PHQ3_Q4
FSS3_Q8
FSS3_Q6

TP4

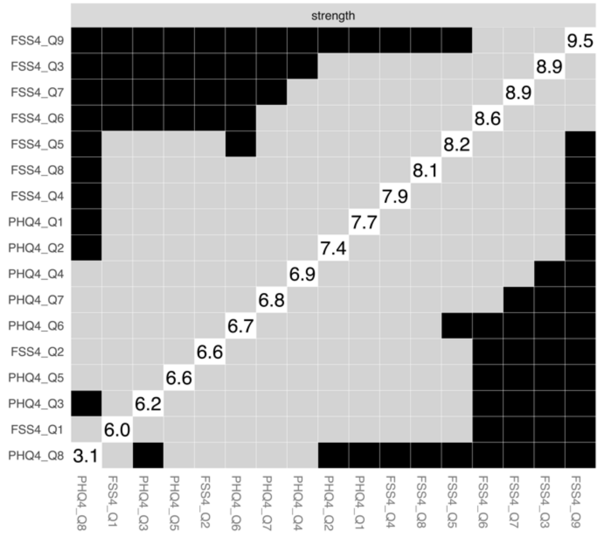

strength
FSS4_Q9
FSS4_Q3
FSS4_Q7
FSS4_Q6
FSS4_Q5
FSS4_Q8
FSS4_Q4
PHQ4_Q1
PHQ4_Q2
PHQ4_Q4
PHQ4_Q7
PHQ4_Q6
FSS4_Q2
PHQ4_Q5
PHQ4_Q3
FSS4_Q1
PHQ4_Q8
9.5
8.9
8.9
8.6
8.2
8.1
7.9
7.7
7.4
6.9
6.8
6.7
6.6
6.6
6.2
6.0
3.1
PHQ4_Q8
FSS4_Q1
PHQ4_Q3
PHQ4_Q5
FSS4_Q2
PHQ4_Q6
PHQ4_Q7
PHQ4_Q4
PHQ4_Q2
PHQ4_Q1
FSS4_Q4
FSS4_Q8
FSS4_Q5
FSS4_Q6
FSS4_Q7
FSS4_Q3
FSS4_Q9

TP5

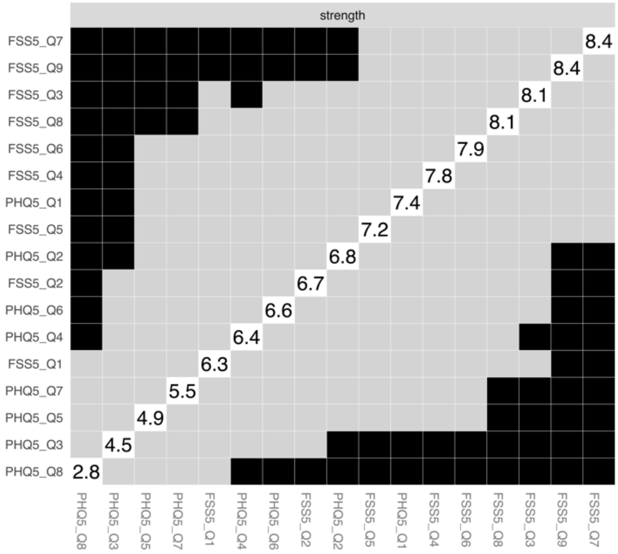

strength
FSS5_Q7
FSS5_Q9
FSS5_Q3
FSS5_Q8
FSS5_Q6
FSS5_Q4
PHQ5_Q1
FSS5_Q5
PHQ5_Q2
FSS5_Q2
PHQ5_Q6
PHQ5_Q4
FSS5_Q1
PHQ5_Q7
PHQ5_Q5
PHQ5_Q3
PHQ5_Q8
8.4
8.4
8.1
8.1
7.9
7.8
7.4
7.2
6.8
6.7
6.6
6.4
6.3
5.5
4.9
4.5
2.8
PHQ5_Q8
PHQ5_Q3
PHQ5_Q5
PHQ5_Q7
FSS5_Q1
PHQ5_Q4
PHQ5_Q6
FSS5_Q2
PHQ5_Q2
FSS5_Q5
PHQ5_Q1
FSS5_Q4
FSS5_Q6
FSS5_Q8
FSS5_Q3
FSS5_Q9
FSS5_Q7

Table 2. Linear models predicting baseline cognitive scores from FSS scores and age

		FSS		Age	
	R^2	β	*p*	β	*p*
MoCA	0.185	-0.12	.185	.297	<.001
CFQ†	0.188	0.46	<.001	0.156	.180
CVLT trial 1	0.243	-0.02	.243	.799	<.001
CVLT total recall	0.237	-0.10	.237	.359	<.001
Stroop color	0.124	0.14	.124	.263	.001
Stroop word	0.140	0.21	.140	.085	.001
Stroop inhibition	0.140	0.09	.140	.436	<.001
Stroop inhibition switch	0.357	0.03	.357	.778	<.001

† Cognitive failures questionnaire
